# *ACSL6* Is Associated with the Number of Cigarettes Smoked and Its Expression Is Altered by Chronic Nicotine Exposure

**DOI:** 10.1371/journal.pone.0028790

**Published:** 2011-12-20

**Authors:** Jingchun Chen, Darlene H. Brunzell, Kia Jackson, Andrew van der Vaart, Jennie Z. Ma, Thomas J. Payne, Richard Sherva, Lindsay A. Farrer, Pablo Gejman, Douglas F. Levinson, Peter Holmans, Steven H. Aggen, Imad Damaj, Po-Hsiu Kuo, Bradley T. Webb, Raymond Anton, Henry R. Kranzler, Joel Gelernter, Ming D. Li, Kenneth S. Kendler, Xiangning Chen

**Affiliations:** 1 Department of Psychiatry and Virginia Institute for Psychiatric and Behavioral Genetics, Virginia Commonwealth University, Richmond, Virginia, United States of America; 2 Department of Pharmacology and Toxicology, Virginia Commonwealth University, Richmond, Virginia, United States of America; 3 Institute for Drug and Alcohol Studies, Virginia Commonwealth University, Richmond, Virginia, United States of America; 4 Interdisciplinary Neuroscience Program, Virginia Commonwealth University, Richmond, Virginia, United States of America; 5 Department of Psychiatry and Neurobehavioral Sciences, University of Virginia, Charlottesville, Virginia, United States of America; 6 Department of Public Health Sciences, University of Virginia, Charlottesville, Virginia, United States of America; 7 Department of Otolaryngology and Communicative Sciences, University of Mississippi Medical Center, Jackson, Mississippi, United States of America; 8 Department of Medicine (Biomedical Genetics), Boston University School of Medicine, Boston, Massachusetts, United States of America; 9 Center for Psychiatric Genetics, NorthShore University HealthSystem Research Institute, Evanston, Illinois, United States of America; 10 Department of Psychiatry and Behavioral Sciences, Stanford University, Stanford, California, United States of America; 11 Biostatistics and Bioinformatics Unit, Medical Resource Council Centre for Neuropsychiatric Genetics and Genomics, Department of Psychological Medicine and Neurology, Cardiff University School of Medicine, Heath Park, Cardiff, United Kingdom; 12 Institute of Epidemiology and Preventive Medicine, National Taiwan University, Taipei, Taiwan; 13 Department of Psychiatry and Behavioral Sciences, Medical University of South Carolina, Charleston, South Carolina, United States of America; 14 Department of Psychiatry, Treatment Research Center, University of Pennsylvania School of Medicine 3900 and Philadelphia Veterans Affairs Medical Center, Philadelphia, Pennsylvania, United States of America; 15 Departments of Psychiatry, Genetics, and Neurobiology, Yale University School of Medicine, New Haven, Connecticut, and Veterans Affairs Connecticut Healthcare Center, West Haven, Connecticut, United States of America; 16 Department of Human and Molecular Genetics, Virginia Commonwealth University, Richmond, Virginia, United States of America; Centre for Addiction and Mental Health, Canada

## Abstract

Individuals with schizophrenia tend to be heavy smokers and are at high risk for tobacco dependence. However, the nature of the comorbidity is not entirely clear. We previously reported evidence for association of schizophrenia with SNPs and SNP haplotypes in a region of chromosome 5q containing the *SPEC2*, *PDZ-GEF2* and *ACSL6* genes. In this current study, analysis of the control subjects of the Molecular Genetics of Schizophrenia (MGS) sample showed similar pattern of association with number of cigarettes smoked per day (numCIG) for the same region. To further test if this locus is associated with tobacco smoking as measured by numCIG and FTND, we conducted replication and meta-analysis in 12 independent samples (n>16,000) for two markers in *ACSL6* reported in our previous schizophrenia study. In the meta-analysis of the replication samples, we found that rs667437 and rs477084 were significantly associated with numCIG (*p* = 0.00038 and 0.00136 respectively) but not with FTND scores. We then used *in vitro* and *in vivo* techniques to test if nicotine exposure influences the expression of *ACSL6* in brain. Primary cortical culture studies showed that chronic (5-day) exposure to nicotine stimulated *ACSL6* mRNA expression. Fourteen days of nicotine administration via osmotic mini pump also increased *ACSL6* protein levels in the prefrontal cortex and hippocampus of mice. These increases were suppressed by injection of the nicotinic receptor antagonist mecamylamine, suggesting that elevated expression of *ACSL6* requires nicotinic receptor activation. These findings suggest that variations in the *ACSL6* gene may contribute to the quantity of cigarettes smoked. The independent associations of this locus with schizophrenia and with numCIG in non-schizophrenic subjects suggest that this locus may be a common liability to both conditions.

## Introduction

Smoking is an important public health problem, and smoking-related diseases take a heavy toll on society. Many studies have shown that smoking is addictive and both genetic and environmental factors influence smoking behaviors. In recent years, with the application of genome-wide association study (GWAS) approach, genetic studies of smoking have made significant progress. One example is the identification of the *CHRNA5/CHRNA3/CHRNB4* locus as a risk factor for smoking quantity [1]–[3]. However, this and other established loci explain only a small proportion of the heritability observed for smoking behaviors. Many more genes remain to be identified for their effects on smoking behaviors.

Smoking is highly prevalent among individuals with a variety of psychiatric disorders [4], [5]. Among them, the comorbidity with schizophrenia is particularly high. Smoking prevalence in those with a schizophrenia diagnosis is 3–4 times higher than that in the general population. Most individuals with schizophrenia smoke more heavily than smokers in the population at large [6]. Several hypotheses have been proposed to explain these phenomena, including a self-medication theory [7], [8]. It is possible that schizophrenic patients smoke to enhance cognition and to inhibit side-effects of neuroleptic drugs, or schizophrenia has an inherited vulnerability to heavy smoking behavior. In a previous study, we found evidence that the *SPEC2*/*PDZ-GEF2*/*ACSL6* locus is associated with schizophrenia [9]. In the present study, in our analyses of smoking phenotypes for the control subjects of the Molecular Genetics of Schizophrenia (MGS) sample, we found substantial association in the acyl CoA synthetase long chain 6 (*ACSL6*) gene with a phenotype based on a categorized number of cigarettes smoked per day (numCIG). To verify if this locus is associated with numCIG, we initiated replication studies using 12 other independent samples.

It is unknown how *ACSL6* variants might affect tobacco smoking and there are no probes available to assess levels of *ACSL6* in the brains of humans. Because nicotine is a major psychoactive ingredient in tobacco that is self-administered in both humans and rodents [10], we used rodent models to determine if chronic nicotine exposure alters *ACSL6* expression in brain areas involved in addictive behaviors and cognition. We conducted *in vitro* and *in vivo* studies, measuring the effects of chronic nicotine exposure on *ACSL6* mRNA expression in rat primary cortical culture and assaying levels of *ACSL6* protein in mouse prefrontal cortex (PFC), hippocampus (HIP), ventral tegmental area (VTA) and nucleus accumbens (NAC) following chronic nicotine exposure in the presence or absence of a nicotinic receptor antagonist, mecamylamine. In this article, we report our findings from these experiments.

## Results

### *ACSL6* association with numCIG in the MGS controls

**Figure 1:**
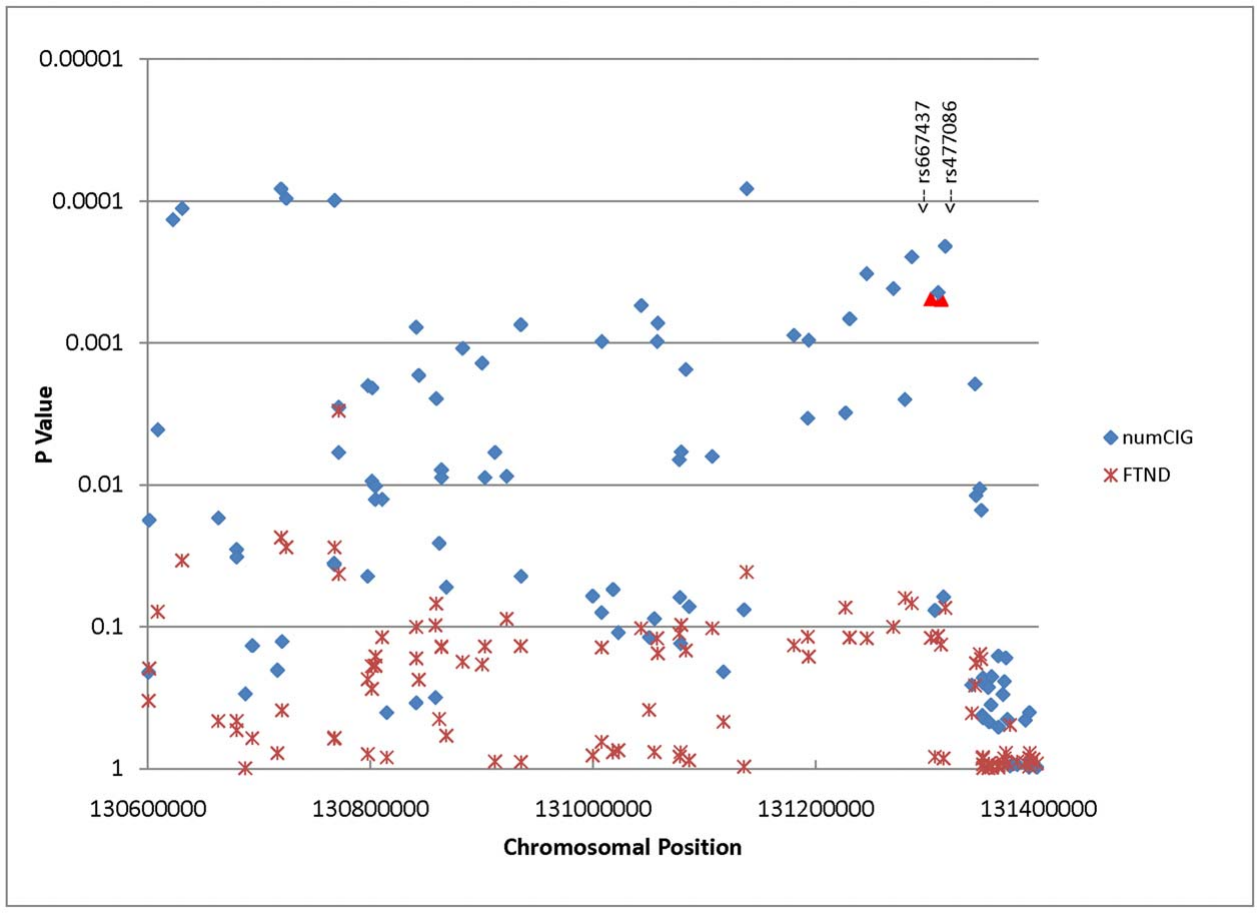
Association analyses of the MGS_EA controls for the *SPEC2*, *PDZ-GEF2*, *FNIP1* and *ACSL6* region. Of the 145 markers tested in this interval, 68 and 12 markers respectively reached nominal significance for numCIG and FTND. The two markers selected for replication were highlighted as red.

In our analysis of the interval covering the *SPEC2*, *PDZ-GEF2* and *ACSL6* genes (about 800 kb), where we reported a long-range haplotype association with schizophrenia [9], 69 of the 145 markers have a *p* value≤0.05 for numCIG, far in excess of chance expectations. In contrast, only 11 markers reach nominal significance for FTND (Figure 1). The lowest *p* value (8×10^−5^) is observed at rs6870930 for numCIG (beta = −0.156, r^2^ = 0.0096), which is located in the *PDZ-GEF2* gene. Of the 24 markers typed in our previous schizophrenia study [9], rs667437 and rs477086 in the *ACSL6* gene are amongst the markers with the lowest *p* values for numCIG (*p* = 0.0005; see Figure 1 and Table 1). However, the association with FTND is not significant. For both markers, the alleles significantly associated with greater number of daily cigarettes smoked (allele G for rs667437 and allele C for rs477086) reside on the risk haplotypes associated with schizophrenia in our previous study.

**Table 1:** *ACSL6* association with numCIG and FTND in the MGS_EA sample.

		numCIG	FTND						
SNP	Effect allele	BETA	SE	STAT	P	BETA	SE	STAT	P
rs667437	A	−0.1351	0.03881	−3.492	**0.0005**	−0.1897	0.1219	−1.557	0.1197
rs477086	T	−0.1351	0.03883	−3.491	**0.0005**	−0.1829	0.1219	−1.501	0.1337

### Replication and meta-analyses of rs667437 and rs477086

**Figure 2:**
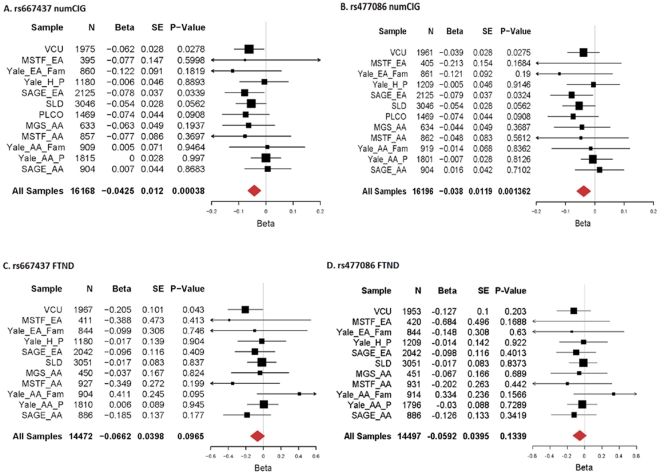
Meta-analyses of rs667437 and rs477086 for numCIG and FTND. A) rs667437, numCIG; B) rs477086, numCIG; C) rs667437, FTND; and D) rs477086, FTND.

We first initiated replication of the finding using our VCU subjects. For rs667437, both numCIG and FTND reach nominal significance (*p* = 0.0262 and 0.0427); for rs477086, although no association is found with FTND (*p* = 0.203), the association with numCIG reaches nominal significance (*p* = 0.0275) (Figure 2A–2D). Following these results, we extended the replication study to other samples, and conducted meta-analyses for all replication samples. Figure 2 shows the results of the meta-analyses. There is no significant heterogeneity for either phenotype across the replication samples (numCIG: rs667437, Q = 7.938, *p* = 0.719; rs477086, Q = 7.865, *p* = 0.725; FTND: rs667437, Q = 9.192, *p* = 0.513, rs477086, Q = 6.052, *p* = 0.811). For both markers, numCIG results are significant (rs667437, *z* = −3.56, *p* = 3.8×10^−4^; rs477086, *z* = −3.20, *p* = 0.001362). Similar to the results we observed in our VCU subjects, the results for FTND are not significant (rs667437, *z* = −1.66, *p* = 0.097; rs477086, *z* = −1.50, *p* = 0.134).

### Nicotine stimulates *ACSL6* mRNA expression in rat cortical culture

**Figure 3:**
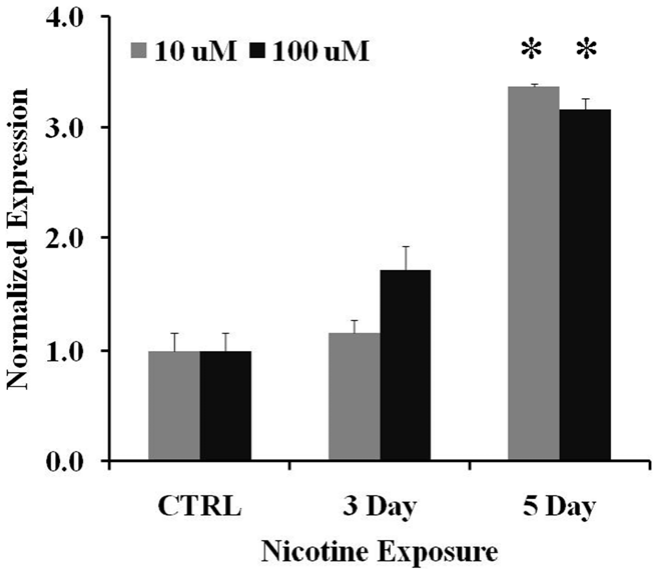
*ACSL6* mRNA expression in rat cortical primary culture after nicotine treatment. The expression levels of *ACSL6* mRNA were significantly increased by 5 days of nicotine exposure for both doses. *p<0.01 compared to saline controls; n = 3. mRNAs were measured by real-time-PCR, and were presented as normalized ΔCt values. Bars represent ± SD.

To follow up the finding that *ACSL6* is associated with the number of cigarettes smoked, we next questioned if nicotine in tobacco might affect expression of *ACSL6* in the central nervous system. We used animal models to determine whether nicotine affects the expression of *ACSL6* in comparative rodent brain areas thought to regulate nicotine/tobacco use and involved in schizophrenia. We first conducted chronic nicotine stimulation experiments in primary rat cortical cultures using 10 and 100 µM nicotine, and measured the mRNA expression of *ACSL6* by real-time quantitative PCR. There is a main effect of nicotine exposure on mRNA expression of *ACSL6* (*F*_2, 6_ = 78.844, *p*<0.001; Figure 3). A *post hoc* t-test reveals that on day 5, the mRNA expression in the nicotine-treated samples is significantly higher than that in the saline controls independent of the concentration of nicotine (*t*_8_ = −3.579, *p*<0.01). The effects on days between the doses are marginal (*t*_8_ = 2.293, *p* = 0.051 for day 3 and *t*_8_ = 2.278, *p* = 0.052 for day 5). These results suggest that nicotine stimulates *ACSL6* mRNA expression in cortical cells. Rat *GAPDH* and TATA box binding protein (*TBP*) primers were used as internal controls. Results from *GAPDH* and *TBP* did not differ significantly and therefore, only *TBP* data was shown (Figure 3).

### Nicotine increases *ACSL6* protein levels in mouse prefrontal cortex (PFC) and hippocampus (HIP)

**Figure 4:**
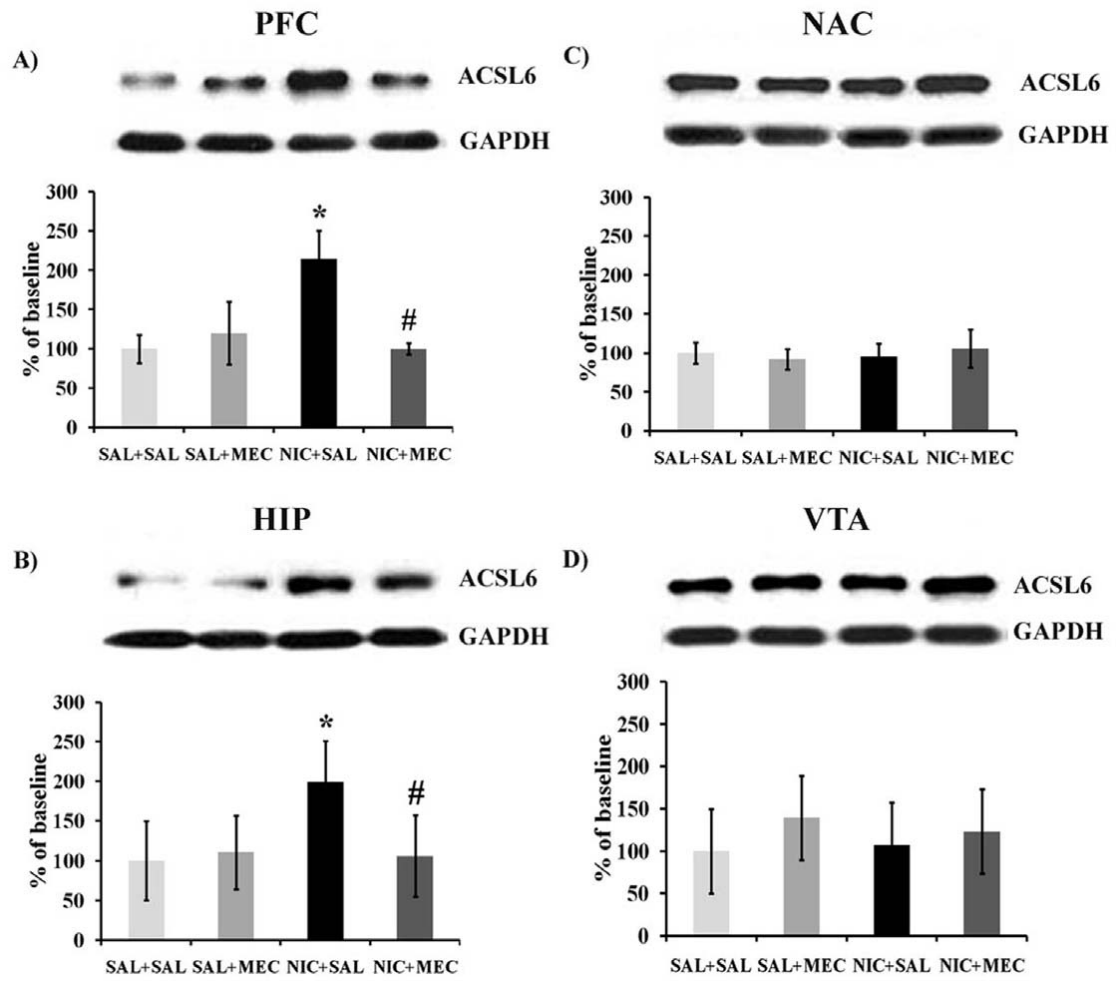
Western blot analyses of *ACSL6* protein in mouse brain regions after chronic nicotine exposure and mecamylamine challenge. Nicotine (NIC+SAL) led to a significant increase in *ACSL6* protein levels in A) the prefrontal cortex and B) the hippocampus [*p<0.01 compared to saline controls (SAL+SAL)]. These increases were suppressed by the administration of mecamylamine (NIC+MEC; # *p*<0.01 compared to nicotine treated NIC-SAL mice). There was no effect of nicotine on levels of *ACSL6* in C) the NAC and D) VTA. (n sizes = 3–8 per group).

We further tested whether nicotine alters *ACSL6* protein expression following *in vivo* nicotine exposure. We chronically administrated a dosing regimen of 36 mg/kg/day nicotine via mini-pumps for 14 days that has been shown to result in dependence-like behavior in mice [11], [12]. On day 15, mice received a challenge s.c. injection of 1 mg/kg mecamylamine or saline vehicle 30 min prior to brain harvest. Four brain areas (PFC, HIP, NAC and VTA) were dissected and processed for Western blotting to quantify the expression levels of *ACSL6* protein. The results are shown in Figure 4. There is a significant interaction of nicotine pretreatment with mecamylamine challenge in the PFC (Figure 4A, *F*_1,11_ = 16.420, *p* = 0.004) and HIP (Figure 4B, *F*_1,27_ = 8.290, *p* = 0.008), but not in the NAC (Figure 4C) and VTA (Figure 4D) after chronic nicotine treatment. *Post hoc* t-tests reveal that animals receiving chronic nicotine show a significant increase in *ACSL6* protein expression in the PFC (*t*_4_ = 4.872, *p* = 0.008) and HIP (*t*_16_ = 4.583, *p*<0.001) of NIC-SAL compared to SAL-SAL control animals. This was not observed following a withdrawal-precipitating dose of the non-selective nicotinic antagonist, mecamylamine. Mecamylamine treatment alone does not change the expression of *ACSL6* protein in the brain regions tested (*F*'s<1.0 in SAL-SAL compared to SAL-MEC mice), but does reverse nicotine associated increases in *ACSL6*. Mice receiving an injection of mecamylamine following 14 days of chronic nicotine treatment (NIC-MEC) produce significantly less *ACSL6* protein than saline-injected controls (NIC-SAL) in both the PFC (*t*_4_ = 5.38, *p* = 0.006) and HIP (*t*_12_ = 3.221, *p* = 0.007). Together these data suggest that continued activation of nicotinic acetylcholine receptors is necessary for the increases of *ACSL6* protein in these brain regions.

## Discussion

In our analyses of the numCIG and FTND phenotypes in the MGS control subjects, we found that multiple markers in the broad region containing the *SPEC2*, *PDZ-GEF2* and *ACSL6* genes showed substantial association with number of cigarettes smoked. While the association signals in these genes did not reach genome-wide significance, the wide spread association signals in the region are consistent with the patterns we observed in our previous schizophrenia studies. Given the high rate of smoking in schizophrenic patients, we sought to test whether this locus is independently associated with tobacco smoking as measured by numCIG and FTND scores in non-schizophrenic subjects. Of the many markers typed in this region in the MGS control sample, we found two overlap markers in our previous study [9], rs667437 and rs477086. The association signals for numCIG from these two markers are among the most significant SNPs identified and are representative of the region. Therefore, we initiated replication of these two *ACSL6* markers in a large population twin sample selected from our twin studies (VCU subjects). In our VCU subjects, both markers, rs667437 and rs477086, reached nominal significance and the risk alleles are the same as those observed in the MGS control subjects. Encouraged by these results, we recruited participation by investigators with independent samples. Using standard meta-analyses, we demonstrate that both markers are significantly associated with the numCIG phenotype and the FTND phenotype shows a trend in the same direction. As these studies were performed with non-schizophrenic subjects, the associations observed are independent of schizophrenia diagnosis. The observation that the same alleles in *ACSL6* are associated with schizophrenia and with numCIG in non-schizophrenic subjects raises an interesting question as to whether there is a common mechanism underlying these associations. To answer this question, we need a sample with both schizophrenia and cigarettes smoking phenotypes. With such a sample, we could estimate how much of the association with schizophrenia can be accounted for by cigarette smoking, and evaluate the relationship between these two separate associations.

*ACSL6* encodes a key enzyme that activates polyunsaturated long chain fatty acids and is involved in lipid metabolism. Its preferred substrates include arachidonic acid, eicosapentaenoic acid and docosahexaenoic acid [13]. Arachidonic acid is a component of arachidonoyl phosphatidyl choline and arachidonic acid-containing inositol phospholipids, which are the major sources of N-arachidonoyl ethanolamine (anandamide) and 2-arachidonoylglycerol (2-AG) [14]. Anandamide and 2-AG are the major endocannabinoids that bind to the cannabinoid receptor 1, a receptor associated with tobacco smoking and other addictive drugs in humans [15]–[18] and shown to promote nicotine reward in animal model studies [19]–[21]. We have reported that the cannabinoid receptor 1 target is associated with tobacco smoking and nicotine dependence [18]. Others have provided evidence that the endogenous cannabinoid system plays a major role in drug reward and addiction, including nicotine [22]–[28]. Intriguingly, it has been proposed that the endocannabinoid system is involved in the etiology of schizophrenia [29] and cannabis use is considered a risk factor for schizophrenia as well [30]. While the exact role of *ACSL6* in schizophrenia and tobacco addiction is unclear at this time, these data warrant further investigation into the potential interactions of *ACSL6* and the cannibinoid system.

Since nicotine is the major psychoactive ingredient in tobacco and *ACSL6* genotype was associated with number of cigarettes smoked, we sought to characterize if nicotine affects expression of *ACSL6* in brain areas that regulate cognition, attention, and addiction behaviors. Using primary cell culture of rat cortex, we show that nicotine stimulates *ACSL6* mRNA expression after 5 days but not 3 days of nicotine exposure. These data suggest that repeated nicotine exposure is necessary for nicotine-associated changes in *ACSL6*. In rodents, humans and non-human primates, the cortex is thought to regulate executive functions that support working memory, cognition and inhibitory control [31]–[34]. The PFC communicates with the HIP which is important for declarative memory and integration of higher brain signals with information projected from sensory systems. This circuitry is compromised in those with schizophrenia who are thought to smoke in part to enhance cognition [33]. Utilizing a mouse model of nicotine exposure that we have previously shown to produce nicotine dependence-like behavior as measured by tolerance and withdrawal [11], [35], we find that chronic systemic exposure to nicotine increases *ACSL6* protein levels in the PFC and HIP of mice. It is interesting that nicotine does not affect the expression of *ACSL6* in the VTA and NAC, areas of the brain that regulate addiction and reward behavior [36]–[38], but the PFC and HIP that have inputs to the VTA and NAC and are also thought to contribute to tobacco addiction [39]–[43]. Interestingly, the *ACSL6* gene appeared in the list of amphetamine addiction genes by an independent GWAS [44], suggesting that *ACSL6* may be involved in addictive behaviors to drugs other than nicotine. The observation that nicotine-associated increases in *ACSL6* are inhibited by the nicotinic receptor antagonist mecamylamine suggests that sustained increases of *ACSL6* protein require activation of nicotinic receptors. It is not clear if this effect is due to changes in transcription or protein degradation and since mecamylamine blocks all nicotinic receptors in brain, further studies should question which receptor subtypes mediate these effects. Although the mechanism of these findings has yet to be determined, our finding is consistent with several studies that have implicated nicotinic receptors in the etiology of schizophrenia [45]–[47] and the quantity of daily cigarettes smoked [1]–[3], [48].

It is not clear whether nicotine-associated increases in *ACSL6* expression might serve to improve cognition and/or enhance neuroplasticity associated with nicotine dependence in individuals with schizophrenia. Many studies have shown that smoking can improve cognitive function, memory and attention in both normal controls and schizophrenia patients [49]–[52] and the effects of cannabinoids on cognitive function and memory [53], [54] are thought to be mediated in part by the cholinergic system [55], [56]. These findings also warrant future studies to determine if alterations of *ACSL6* in brain might support self-medication, cigarette craving, withdrawal or primary or conditioned reinforcing effects of cigarettes.

In our previous fine-mapping study of schizophrenia, we found that haplotypes spanning *SPEC2*, *PDZ-GEF2* and *ACSL6* genes were associated with the disease [9]. In the current study, we found that this same genomic interval showed a similar pattern of association with numCIG in the MGS controls, an association that we confirmed in a meta-analysis of 12 independent samples of non-schizophrenic subjects. Since the *ACSL6* gene is in high LD with other genes, i.e. *SPEC2* and *PDZ-GEF2*, we cannot exclude the possibility that the observed association signals may reflect the activity of other genes in this region, but our rodent studies suggest that nicotine in tobacco increases expression of *ACSL6* message and protein. Given the heavy tobacco use of individuals diagnosed with schizophrenia, our results raise interesting questions in regard to whether schizophrenic smokers may ingest tobacco to regulate *ACSL6*. In separate studies, several other genes have been associated with both nicotine dependence [18], [20], [57] and schizophrenia [58]–[64]. The *SPEC2*, *PDZ-GEF2* and *ACSL6* region may be another locus that contributes to shared susceptibility to schizophrenia and tobacco addiction. Taken together, these studies provide convergent evidence that tobacco use and schizophrenia may share some common underlying mechanisms and that common vulnerability genes such as *ACSL6* are regulated by nicotinic receptors.

## Materials and Methods

### Human studies

This study was conducted according to the principles expressed in the Declaration of Helsinki. For all human studies, all participants provided written informed consent. The study protocol, forms, and procedures were approved by Institutional Review Boards/Ethics Committees at Virginia Commonwealth University (VCU subjects), Yale University School of Medicine (The Yale/UConn subjects), University of Virginia (MSTF study) and all the other participating Institutional Review Boards.

#### MGS controls

The control subjects for the MGS were a population sample selected for the GWAS of schizophrenia [65]. Subjects were sampled proportionally from 25 major population areas. All subjects completed an online, short, self-report clinical assessment after giving informed consent through an online procedure and prior to venipuncture being arranged. The self-report screen focused on common psychiatric disorders including substance use problems, along with age, sex, height, weight, and the ethnic background of their grandparents. Specific to smoking phenotypes, the subjects were ascertained with the full FTND questionnaire. The information corresponding to the FTND was extracted from the phenotype dataset. FTND scores were obtained based on the answers of the participants to the questionnaires. Subjects who did not smoke a whole cigarette (a “No” answer to the question, “Have you ever smoked a whole cigarette?”) were excluded from FTND score calculation. To be consistent with ascertainment of our VCU subjects, only subjects who answered “Yes” to the question “did you smoke cigarettes on a daily basis” and who smoked ≥5 cigarettes per day were included in the analyses of FTND. The numCIG phenotype was constructed using the raw number of cigarettes smoked per day at the time when the subjects smoked most heavily in their lifetime. Based on the raw data distribution, we divided the subjects into 4 categories using these cut-offs: 0 for those who smoked 1 cigarette per day; 1 for those who smoked 2–14 cigarettes per day; 2 for those who smoked 15–25 cigarettes per day and 3 for those who smoked 26 cigarettes per day or more. For the numCIG phenotype, all subjects reporting smoking were included in the analyses. The MGS controls included both European Americans (MGS_EA) and African Americans (MGS_AA). For this study, there were 1,342 and 1,843 European American (EA) subjects having FTND and numCIG phenotypes respectively, and 454 and 639 AA subjects having FTND and numCIG phenotypes, respectively. The distributions of these phenotypes are shown in Figure S1. The distribution of both phenotypes is normal.

#### The SAGE subjects

The Study of Addiction: Gene and Environment (SAGE) is part of the Gene Environment Association Studies initiative (GENEVA) funded by the National Human Genome Research Institute aiming at understanding the impact of genes and environments on substance dependence and addiction. The SAGE sample consisted of 3 subsamples: the Collaborative Study on the Genetics of Alcoholism (COGA) [66], the Family Study of Cocaine Dependence (FSCD) [67], and the Collaborative Genetic Study of Nicotine Dependence (COGEND) [1]. Although FTND scores were available for all subjects, the raw number of cigarettes smoked per day was not available. Instead, subjects were assigned to one of the four categories based on the FTND questionnaire that grouped individuals by the number of cigarettes smoked per day (subjects were assigned 0 if they smoked 0–10 cigarettes; 1 if they smoked 11–20 cigarettes; 2 if they smoked 21–30 cigarettes and 3 if they smoked more than 30 cigarettes per day). The SAGE sample included 2,125 subjects with self-reported European ancestry (SAGE_EA) and 904 subjects with self-reported African ancestry. For the FSCD subsample, only one subject from each family was used.

#### The Lung cancer and smoking study

This is a GWAS to investigate the genetic determinants of lung cancer risk. This study is also part of the GENEVA initiative and consisted of two samples. The first is the Environment and Genetics in Lung Cancer Etiology Study (EAGLE) [68], a population-based, biologically intensive, case-control study from the Lombardy region of Italy including ∼2,000 newly diagnosed lung cancer cases and ∼2,000 age-, gender- and region- matched controls. This study is also referred to as the Smoking and Lung Disease (SLD) study. The second is the Prostate, Lung, Colon and Ovary (PLCO) [69], [70] Cancer Screening Trial, from which ∼850 lung cancer cases and ∼850 controls matched on age and gender were used. The subjects were ascertained for a variety of smoking phenotypes, including smoking status, persistent smoking, quit attempts, and the Fagerström questionnaire. PLCO participants were all EA and EAGLE included subjects from Italy who were all with European ancestry. EAGLE is a case-control study and includes 3,937 phenotyped subjects. PLCO is a screening trial with a cohort design and includes 1,651 phenotyped subjects.

#### The VCU subjects

The subjects from the Virginia Commonwealth University were selected from the Mid-Atlantic Twin Registry. In this study, we selected regular smokers (defined as those who smoked at least 7 cigarettes per week for a month or more) from our population twin studies [71]–[73]. Tobacco smoking and nicotine dependence were assessed by the Fagerström Tolerance Questionnaire (FTQ) and/or FTND [74], [75]. All regular smokers with DNA samples were included except when self-reported ancestry was not Caucasian. One of the co-twins was selected randomly for inclusion. The final sample included 2,138 individuals, with 1,438 males and 700 females. All subjects were aged 18 to 65 at the time of FTND ascertainment and all self-reported being of European ancestry.

#### Mid-South Tobacco Family (MSTF) study

The subjects of the MSTF study with either AA or EA origin were recruited primarily during 1,999–2,004 from the Mid-South states of Tennessee, Mississippi, and Arkansas. Proband smokers were required to be at least 21 years old, to have smoked for at least 5 years, and to have consumed at least 20 cigarettes per day for the last 12 months. Siblings and biological parents of a proband smoker were recruited whenever possible, regardless of their smoking status. The study includes 2,037 participants in 602 nuclear families, with 671 subjects in 200 EA families and 1,366 subjects in 402 AA families. The degree of nicotine dependence of each smoker was ascertained by the three most commonly used measures: Smoking Quantity (SQ; defined as the number of cigarettes smoked per day), the Heaviness of Smoking Index (HSI; 0–6 scale), and the FTND score (0–10 scale). All three measures have been used consistently in previous reports on nicotine dependence in this sample [57], [76]–[78]. In this study, only the FTND scores and numCIG phenotypes were used.

#### The Yale/UConn subjects

The subjects involved in the Yale/UConn study were both families and unrelated individuals. The family samples were recruited from several clinical sites (principally from Yale University School of Medicine and the University of Connecticut Health Center, but also from McLean Hospital and the Medical University of South Carolina) through siblings meeting DSM-IV criteria for cocaine dependence or opioid dependence. The sample of unrelated individuals was ascertained as cases affected for cocaine, opioids, or alcohol dependence and screened controls. The Yale/UConn family sample included 2,129 AAs (including 132 self-reported Hispanics) and 1,706 EAs (including 310 self-reported Hispanics) [79]. Of these, 1,858 subjects from 893 families had genotype and phenotype data. The Yale/UConn case control sample included 1,912 AAs (including 76 Hispanics) and 1,476 EAs (including 176 Hispanics).

#### Marker Selection, genotyping and genotype imputation

Since our initial goal was to test whether markers associated with schizophrenia were also associated with smoking and nicotine dependence phenotypes, we compared the markers from our schizophrenia study with those included in the Affymetrix 6.0 chipset (the MGS samples were first genotyped with this chipset) in the *PDZ-GEF2*/*ACSL6* region. We found that only 2 of the markers (rs667437 and rs477086) used in our previous schizophrenia study were in the 6.0 chipset. Although the association of these two markers with schizophrenia was not among the strongest observed in the schizophrenia study, they were among the best results in our initial association analyses of the MGS sample. Therefore, only these 2 markers were selected for the present study.

For the GWAS datasets (MGS, SAGE and Lung Cancer), DNA preparation and genotyping have been reported previously. The MGS subjects were typed using an Affymetrix platform (SNP 6.0), and the SAGE and Lung Cancer subjects were typed using an Illumina platform (Human 1 M-Duo). For these datasets, we accepted the quality filtering procedures of each individual study and used the genotypes directly after download. The VCU subjects, the Yale/UConn and MSTF subjects were genotyped by each group using the TaqMan method. For the 2 SNPs used in this study, the assays were designed and synthesized by Applied BioSystems (Foster city, CA, USA). Standardized procedures recommended by the manufacturer were used. The Illumina marker set included only one of the two SNPs used in this study: rs477086. The genotypes for rs667437 were imputed using the fastPHASE program [80] with the MGS_EA or MGS_AA as a reference panel for the EA and AA samples respectively. Imputations were also conducted with HapMap subjects as references, and the imputed genotypes were almost identical to those using MGS samples as references.

#### Association and meta-analysis

Association analyses were performed for each sample with the PLINK software package [81]. We used two phenotypes, FTND score and numCIG, where numCIG was a categorical phenotype. Both phenotypes were treated as quantitative traits in linear regression. For population samples, we used linear regression with sex, age and ethnicity (Hispanics) as covariates. For family samples, we used the QFAM module and the within-family statistics (MSTF, Yale_EA_Fam and Yale_AA_Fam samples). For GWAS datasets, we adapted the principle components used in the original studies. Meta-analyses were performed with the GWAMA software package [82] for replication samples only. Summary statistics (beta, se and sample size) were extracted from individual analyses and used in the meta-analyses. The package can perform both fixed effect and random effect meta-analyses. In our analyses, since the heterogeneity tests (both Cochran's Q statistics and *I^2^*) were non-significant, we reported the results from the fixed effect analyses. Meta-analysis results were plotted with the R package rmeta.

### Animal studies

The experimental protocol was approved by the Institutional Animal Care and Use Committee at Virginia Commonwealth University (the University Animal Welfare Assurance Number: A3281-01), and all animals were treated according to the *Guidelines for the Care and Use of Laboratory Animals*, as set forth by the National Institutes of Health. Animals were maintained on a 12-hour light/dark cycle in a temperature (21°C) and humidity controlled vivarium with *ad libitum* access to food and water. Experiments were performed during the light cycle.

#### Rat *in vitro* expression study

Sprague–Dawley timed-pregnant rats were obtained from Zivic Laboratories (Allison Park, PA, USA). On postnatal day 1, the litter was transferred together to the laboratory where brain harvests took place. Mixed neuronal plus glial cultures were prepared as described previously [83], [84]. Briefly, the cortex of 4–5 pups was dissected in a sterile saline solution (137 mM NaCl, 5.3 mM KCl, 0.17 mM Na_2_HPO_4_•7H_2_O, 0.22 mM KH_2_PO_4_ and 0.0012 g/L Phenol Red) under a laminar flow hood then transferred into a sterile-filtered 0.1% porcine trypsin dissection solution, minced and incubated at room temperature for 10 min. Brain sections were rinsed twice in plating medium (DMEM, 10% FBS, 1% L-glutamine and 1% Penicillin/Streptomycin) to stop the trypsin reaction, triterated with a cotton-plugged glass pipette, spun 7 min at 1,000 RPM with medium replaced and then poured through a 70 µm nylon cell filter (BD Falcoln, Bedford, MA, USA) for plating. 1.7×10^6^ cells were seeded into each well of a 6-well plate. Cells were cultured in a 5% CO_2_ incubator at 37°C. Three days after plating, and every 3 days thereafter, 1 mL medium was removed from each well and replaced with 1 mL of fresh mixed growth medium containing minimal essential medium, 10 mM glucose, penicillin 100 U/mL, streptomycin 100 µg/mL, and 5% horse serum. On day 14, *in vitro* media was removed and 2.0 mL media was quickly replaced to ensure a consistent stimulating volume. Cells treated with 0 µM, 10 µM, and 100 µM Nicotine ((-)-Nicotine hydrogen tartrate salt, dissolved in 0.9% sodium chloride) were cultured for another 3 and 5 days. At each time point, the media was removed, wells were washed twice with PBS, and cells were harvested in 200 µl PBS. Cells from three wells were pooled into an Eppendorf tube. Total RNA was extracted from cells using TRIzol Reagent (Invitrogen, Eugene, OR, USA) and quantified by Quant-iT™ RNA Assay Kit (Invitrogen, Eugene, OR, USA). cDNA was synthesized using 2 µg of total RNA and 50 ng of random hexamers according to the first-strand cDNA synthesis protocol provided with SuperScript III RNase H-Reverse Transcriptase (Invitrogen, Eugene, OR, USA). For real time-PCR, samples were analyzed in triplicate 20 µl reactions including 25 ng of cDNA, 250 nmol of primer, 1× PCR buffer, 2 mM MgCl2, 0.08 mM dNTPs, 0.01 u/µl Taq polymerase (Invitrogen, Eugene, OR, USA) and 1/10 X SyBR® Green (Sigma, St. Louis, MO, USA). Primers were designed to coding sequence using Primer3 (v. 0.4.0). PCR reactions using rat TATA box binding protein (*TBP*) and *GAPDH* primers were used as internal controls. The Primer sequences for the *ACSL6*, *TBP* and *GAPDH* are as follows: *ACSL6* forward, 5′-TTTCACGAGCGGTACAACAG-3′, *ACSL6* reverse, 5′-GTGTACATCCGCACAAGTGG -3′; *TBP* forward, 5′-TATAATCCCAAGCGGTTTGC-3′, *TBP* reverse, 5′-CAGCCTTATGGGGAACTTCA-3′; *GAPDH* forward, 5′-AAGGGCTCATGACCACAGTC-3′, *GAPDH* reverse, 5′-CAACGGATACATTGGGGGTA-3′. PCR was conducted and the expression level of each reaction was determined by the C_T_ value. The results from three replicated assays were averaged to produce a single mean C_T_ value for each treatment condition. The relative expression level between *ACSL6* and *TBP* or *GAPDH* for each condition was calculated by the 2^−ΔCT^ method, where Δ*C*_T_ = C_T_ *^ACSL6^*−C_T_ *^TBPor GAPDH^* [85].

#### Mouse *in vivo* expression study

Male 129SvJ mice were purchased from Jackson Laboratories (Bar Harbor, ME). Animals were 8–10 weeks of age at the start of the studies. Mice were anesthetized with sodium pentobarbital (45 mg/kg by intraperitoneal injection) and implanted subcutaneously (s.c.) with Alzet osmotic mini pumps [model 2,004, Durect Corporation, Cupertino, CA, USA] filled with (-)-nicotine (NIC) or saline (SAL) solution as described in Jackson et al. [11]. The concentration of nicotine was adjusted according to animal weight and mini pump flow rate so that mice were infused with 36 mg/kg/day for 14 days. The dose and duration of nicotine exposure were chosen based on previous behavioral studies [11], [12] which show that significant tolerance and nicotine withdrawal signs are produced in mice after this treatment regimen. On the morning of Day 15, chronic nicotine- and saline-infused mice were injected s.c. with 1.0 or 2.0 mg/kg of a non-selective nicotinic receptor antagonist, mecamylamine (MEC) or saline vehicle (SAL) followed by a 30 min waiting period. There was no significant difference in *ACSL6* protein levels between the 1 and 2 mg/kg doses of mecamylamine used across cohorts of animals, so these data were combined to create 4 experimental treatment groups (SAL+SAL, SAL+MEC, NIC+SAL, NIC+MEC). Mice were then euthanized by cervical dislocation. Brain sections were dissected and placed immediately in cold extraction buffer for dissection of PFC, HIP, NAC and VTA. Brain tissues were dissected and homogenized as described previously [86]. Protein concentrations were determined using the *DC* protein assay (Bio-Rad Laboratories, Hercules, CA, USA), and 30 µg of protein were mixed with 6× blue gel loading dye (New England Biolabs, Ipswich, MA, USA) and heated for 5 minutes at 95°C. Samples were then separated by SDS-polyacrylamide gel electrophoresis on a 10% Tris-HCL gel and subjected to immunoblotting with anti-*ACSL6* antibody (1∶500 from goat; sc-48005, Santa Cruz Biotechnology, Inc., Santa Cruz, CA, USA) or anti-*GAPDH* (1∶50,000 from mouse; Advanced ImmunoChemical Ins., Long Beach, CA, USA) primary antibodies overnight at 4°C. Blots were rinsed in TBST and then incubated in secondary antibodies (1∶2000 anti-goat or 1∶50,000 mouse, Vector Laboratories, Inc., Burlingame, CA, USA) for 1 hour at room temperature followed by three times of TBST 10 min washes. Specific bands were detected by enhanced chemiluminesence (GE Healthcare Bio-Sciences, Piscataway, NJ, USA), exposed to X-Ray film, and quantified using Image J software (Rasband WS, National Institutes of Health, Bethesda, MD; http://rsb.info.nih.gov/ij/, 1997–2006). *ACSL6* protein levels were normalized against *GAPDH* for loading control and against vehicle-treated control subjects to enable comparisons across blots.

## Supporting Information

Figure S1 **Distribution of number of cigarettes smoked per day (A) and FTND scores in the MGS control subjects (B).**
(DOCX)
