# Correction: *ACSL6* Is Associated with the Number of Cigarettes Smoked and Its Expression Is Altered by Chronic Nicotine Exposure

**DOI:** 10.1371/annotation/071abcbd-6f0c-4c4b-9507-dae42450457e

**Published:** 2012-08-14

**Authors:** Jingchun Chen, Darlene H. Brunzell, Kia Jackson, Andrew van der Vaart, Jennie Z. Ma, Thomas J. Payne, Richard Sherva, Lindsay A. Farrer, Pablo Gejman, Douglas F. Levinson, Peter Holmans, Steven H. Aggen, Imad Damaj, Po-Hsiu Kuo, Bradley T. Webb, Raymond Anton, Henry R. Kranzler, Joel Gelernter, Ming D. Li, Kenneth S. Kendler, Xiangning Chen

The following information was missing from the Acknowledgements section: 

This study also makes use of data generated by Genome-Wide Association of Schizophrenia Study (PI: Pablo V. Gejman). Funding support for schizophrenia GWAS was provided by the National Institute of Mental Health (R01 MH67257, R01 MH59588, R01 MH59571, R01 MH59565, R01 MH59587, R01 MH60870, R01 MH59566, R01 MH59586, R01 MH61675, R01 MH60879, R01 MH81800, U01 MH46276, U01 MH46289 U01 MH46318, U01 MH79469, and U01 MH79470) and the genotyping of samples was provided through the Genetic Association Information Network (GAIN). The datasets used for the analyses described in this manuscript were obtained from the database of Genotypes and Phenotypes (dbGaP) found at http://www.ncbi.nlm.nih.gov/gap [^] through dbGaP accession number phs000021.v3.p2 and phs000167v1.p1. The SAGE samples (PI: Laura J. Bierut) were GWAS datasets sponsored by the National Human Genome Research Institute. Funding support for the SAGE Study was provided through the NIH Genes, Environment and Health Initiative [GEI] (U01 HG004422). Funding support for genotyping, which was performed at the Johns Hopkins University Center for Inherited Disease Research, was provided by the NIH GEI (U01HG004438), the National Institute on Alcohol Abuse and Alcoholism, the National Institute on Drug Abuse, and the NIH contract "High throughput genotyping for studying the genetic contributions to human disease" (HHSN268200782096C). The datasets used for the analyses described in this manuscript were obtained from dbGaP at http://www.ncbi.nlm.nih.gov/projects/gap/cgibin/study.cgi?study_id=phs000092.v1.p1 [^] through dbGaP accession number phs000092.v1.p. The SLD sample (also called EAGLE study, PI: Neil Caporaso) and the PLCO sample (PI: Maria Teresa Landi) were GWAS datasets sponsored by the National Cancer Institute for the study of genome wide scan for lung cancer and smoking. Funding support for the GWAS of Lung Cancer and Smoking was provided through the NIH Genes, Environment and Health Initiative [GEI] (Z01 CP 010200). Funding support for genotyping, which was performed at the Johns Hopkins University Center for Inherited Disease Research, was provided by the NIH GEI (U01HG004438).The datasets used for the analyses described in this manuscript were obtained from dbGaP at

http://www.ncbi.nlm.nih.gov/gap [^] through dbGaP accession number phs000093. The funders had no role in study design, data collection and analysis, decision to publish, or preparation of the manuscript. 

