# Supplementary material for: ACSL6 Is Associated with the Number of Cigarettes Smoked and Its Expression Is Altered by Chronic Nicotine Exposure
Source: PLoS One. 2011 Dec 20;6(12):e28790. doi: 10.1371/journal.pone.0028790 (PMC3243669; doi:10.1371/journal.pone.0028790)
Supplement: Figure S1 — Distribution of number of cigarettes smoked per day (A) and FTND scores in the MGS control subjects (B). (DOCX) [file pone.0028790.s001.docx]

Figure S1. Distribution of number of cigarettes smoked per day (A) and FTND scores in the MGS control subjects (B).
